# Individual and population-scale carbon and nitrogen isotopic values of *Procambarusclarkii* in invaded freshwater ecosystems

**DOI:** 10.3897/BDJ.10.e94411

**Published:** 2022-10-20

**Authors:** Cristina Di Muri, Paloma Alcorlo, Roberta Bardelli, Jordi Catalan, Esperança Gacia, Maria Teresa Guerra, Ilaria Rosati, David X. Soto, Salvatrice Vizzini, Giorgio Mancinelli

**Affiliations:** 1 LifeWatch ERIC, Lecce, Italy LifeWatch ERIC Lecce Italy; 2 Italian National Research Council (CNR), Institute of Research on Terrestrial Ecosystems (IRET), Lecce, Italy Italian National Research Council (CNR), Institute of Research on Terrestrial Ecosystems (IRET) Lecce Italy; 3 Universidad Autónoma de Madrid, Madrid, Spain Universidad Autónoma de Madrid Madrid Spain; 4 Biodiversity and Global Change Research Center (CIBC), Madrid, Spain Biodiversity and Global Change Research Center (CIBC) Madrid Spain; 5 University of Palermo, Palermo, Italy University of Palermo Palermo Italy; 6 Centre for Research on Ecology and Forestry Applications (CREAF), Barcelona, Spain Centre for Research on Ecology and Forestry Applications (CREAF) Barcelona Spain; 7 Spanish National Research Council (CSIC), Barcelona, Spain Spanish National Research Council (CSIC) Barcelona Spain; 8 Centre for Advanced Studies of Blanes (CEAB), Spanish National Research Council (CSIC), Girona, Spain Centre for Advanced Studies of Blanes (CEAB), Spanish National Research Council (CSIC) Girona Spain; 9 University of Salento, Lecce, Italy University of Salento Lecce Italy; 10 LifeWatch Italy, Lecce, Italy LifeWatch Italy Lecce Italy; 11 National Interuniversity Consortium for Marine Sciences (CoNISMa), Roma, Italy National Interuniversity Consortium for Marine Sciences (CoNISMa) Roma Italy; 12 Italian National Research Council (CNR), Institute of Marine Biological Resources and Biotechnologies (IRBIM), Lesina, Italy Italian National Research Council (CNR), Institute of Marine Biological Resources and Biotechnologies (IRBIM) Lesina Italy

**Keywords:** invasive species, Louisiana crayfish, red swamp crayfish, stable isotopes, ecological impact

## Abstract

**Background:**

Freshwater ecosystems are amongst the most threatened habitats on Earth; nevertheless, they support about 9.5% of the known global biodiversity while covering less than 1% of the globe’s surface. A number of anthropogenic pressures are impacting species diversity in inland waters and, amongst them, the spread of invasive alien species is considered one of the main drivers of biodiversity loss and homogenisation in freshwater habitats.

Crayfish species are widely distributed freshwater invaders and, while alien species introductions occur mostly accidentally, alien crayfish are often released deliberately into new areas for commercial purposes. After their initial introduction, crayfish species can rapidly establish and reach high-density populations as a result of their adaptive functional traits, such as their generalist diet.

The Louisiana crayfish *Procambarusclarkii* (Girard, 1852) is globally considered one of the worst invaders and its impact on recipient freshwater communities can vary from predation and competition with native species, to modification of food webs and habitat structure and introduction of pathogens. Native to the south United States and north Mexico, *P.clarkii* has been introduced in Europe, Asia and Africa, determining negative ecological and economic impacts in the majority of invaded habitats where it became dominant within the receiving benthic food webs. Due to its flexible feeding strategy, *P.clarkii* exerts adverse effects at different trophic levels, ultimately affecting the structure and dynamics of invaded food webs. It is, therefore, paramount to evaluate the ecological consequences of *P.clarkii* invasion and to quantify its impact in a spatially explicit context.

**New information:**

In the past decades, the analysis of stable isotopes of carbon, nitrogen and other elements has become a popular methodology in food web ecology. Notably, stable isotope analysis has emerged as a primary tool for addressing applied issues in biodiversity conservation and management, such as the assessment of the trophic ecology of non-indigenous species in invaded habitats. Here, we built two geo-referenced datasets, resolved respectively at the population and individual scale, by collating information on δ^13^C and δ^15^N values of *P.clarkii* within invaded inland waters. The population-scale dataset consists of 160 carbon and nitrogen isotopic values of the Louisiana crayfish and its potential prey, including living and non-living primary producers and benthic invertebrates. The dataset resolved at individual scale consists of 1,168 isotopic records of *P.clarkii*. The isotopic values included within the two datasets were gathered from 10 countries located in Europe, Asia, Africa and North America, for a total of 41 studies published between 2005 and 2021. To the best of the authors’ knowledge, this effort represents the first attempt to collate in standardised datasets the sparse isotopic information of *P.clarkii* available in literature. The datasets lend themselves to being used for providing a spatially explicit resolution of the trophic ecology of *P.clarkii* and to address a variety of ecological questions concerning its ecological impact on recipient aquatic food webs.

## Introduction

Crayfish species are amongst the most successful and widespread freshwater invaders worldwide (Capinha et al. 2010, Gallardo et al. 2015). The crayfish ability for acting as keystone consumers feeding on a variety of trophic levels is one of the adaptive traits that lead the successful establishment of these crustaceans in non-native habitats (Lodge et al. 1994, Nyström et al. 1996). Invasive crayfish can determine trophic cascades and adverse effects on energy fluxes in invaded habitats and these crustaceans are often the largest and longest-living invertebrates of benthic food webs (Gherardi et al. 2011). The global distribution of crayfish species was once uneven, with 75% of the total number of species native to North and Central America (Gherardi 2007). Nowadays, owing to globalisation and international trade, an increasing number of crayfish species is widely distributed across continents as a consequence of both accidental human-mediated translocations and intentional introductions for commercial purposes (Lodge et al. 2012).

After the initial introduction, crayfish can quickly spread and colonise new habitats thanks to their dispersal ability, including the capacity of some species to travel long distances and even overland at times (Souty-Grosset et al. 2016), such as in the case of the Louisiana crayfish (or red swamp crayfish) *Procambarusclarkii* (Girard, 1852). Similarly to other crayfish, *P.clarkii* has a flexible diet and it can also tolerate a broad range of environmental conditions including extreme variations in oxygen level, water salinity and acidity and prolonged droughts (Alcorlo et al. 2004, Loureiro et al. 2015). Despite the relatively small native range, encompassing the southern USA and north-eastern Mexico, *P.clarkii* has colonised all continents, except Australia and Antarctica and it is globally considered one of the worst invasive species (Loureiro et al. 2015). Due to its economic importance, *P.clarkii* has been deliberately introduced in different countries for aquaculture and fishing activities and it became invasive for its capacity of rapidly colonising new areas reaching high population densities (Hänfling et al. 2011).

The impact of *P.clarkii* spans from predation and competition with native species, to disruption of food webs and habitat structure and introduction of pathogens (Souty-Grosset et al. 2016). Introduced populations of the Louisiana crayfish have determined a reduction of freshwater biodiversity with a negative impact on several taxonomic groups such as amphibians (Ficetola et al. 2011, Ficetola et al. 2012), macroinvertebrates (Correia and Anastácio 2008) and macrophytes (Carreira et al. 2014). Furthermore, *P.clarkii* is one of the vectors of the crayfish plague, which is lethal for freshwater crayfish from regions outside North America and has already determined a number of local extinctions in Europe (Bland 2017, Manenti et al. 2018, Martín-Torrijos et al. 2019). The economic impact associated with the management and control of introduced populations of *P.clarkii* is also considerable for the agricultural and fishery sectors (de Moor 2002, Anastacio et al. 2005).

As an opportunistic feeder (Alcorlo and Baltanás 2013), but also as an important prey item of freshwater top predators (Soto et al. 2016), *P.clarkii* is expected to play a crucial role in shaping the structure and dynamics of invaded aquatic food webs (Alcorlo and Baltanás 2013). Traditionally, stomach content analyses have been used to investigate the trophic habits of the species and its adaptation strategy to newly-invaded environments (Hyslop 1980). Over the last three decades, stable isotope analysis of nitrogen and carbon (and more recently of hydrogen; Soto et al. 2011) has been proven as an extremely useful methodology in trophic ecology because it can provide quantitative, standardised and reliable information on the dietary habits of species - including bioinvaders - and, consequently, it can be used to estimate the trophic impact of invasive species on local communities (Vander Zanden et al. 1999, Rush et al. 2012, Mancinelli and Vizzini 2015).

## General description

### Purpose

The two datasets presented herein collate geo-referenced δ^13^C and δ^15^N values of *P.clarkii* and its potential prey in invaded inland and brackish waters. The dataset resolved at population scale includes mean values and standard deviations of δ^13^C and δ^15^N for the Louisiana crayfish populations and their potential animal and vegetal prey. The individual-scale dataset collates isotopic values of single specimens of *P.clarkii*, similarly to the dataset with stable isotopes of the Atlantic blue crab *Callinectessapidus* published by Di Muri et al. (2022). In the two datasets, isotopic values are expressed in standard delta notation (permil [‰]), indicating the deviation from atmospheric nitrogen and from Vienna Pee Dee Belemnite (VPDB) limestone, respectively, as scale-defining standards for nitrogen and carbon (Hood-Nowotny and Knols 2007). Specifically, δ^15^N or δ^13^C values are calculated as [(R_Sample_/R_Standard_) – 1] × 1000, where R is the ratio of the heavy vs. the light isotope (i.e. ^15^N/^14^N or ^13^C/^12^C).

The two datasets can be used for a variety of comparative analyses including the calculation of the trophic position of the Louisiana crayfish (population-scale dataset) and the calculation of metrics and descriptors of its isotopic niche (individual-scale dataset) accounting for isotopic differences in the baseline at each location. Both are examples of input files used for the Crustaceans workflow of the LifeWatch ERIC Internal Joint Initiative. The analytical workflow aims at identifying regional-scale climatic predictors of the trophic position of the two model invasive crustaceans, i.e. *P.clarkii* and the Atlantic blue crab *C.sapidus*.

## Project description

### Title

LifeWatch ERIC Internal Joint Initiative - Functional biogeography of invaders: the case of two widely-distributed omnivorous crustaceans (https://bit.ly/iji-crustaceans)

### Personnel

Cristina Di Muri, Giorgio Mancinelli, Ilaria Rosati, Lucia Vaira

### Study area description

**Figure 1.**
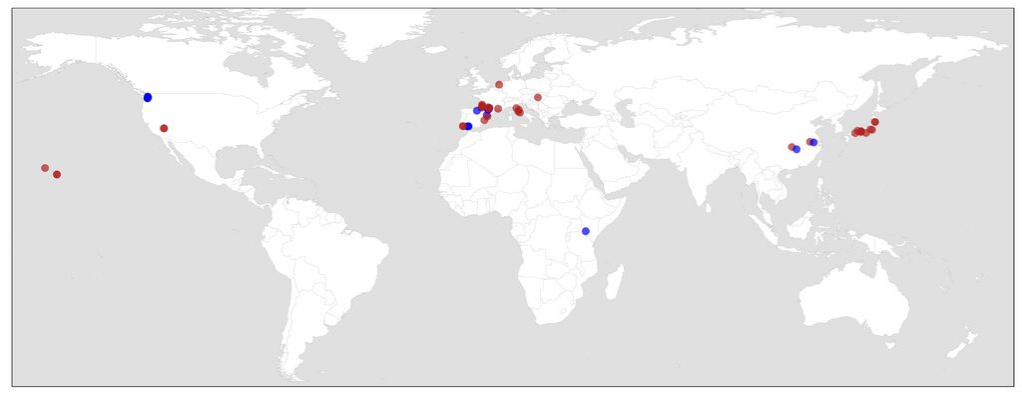
Distribution map of the locations included in the datasets. The locations represent the study sites where the population- and individual-scale isotopic values of *Procambarusclarkii* and its potential prey were collated (in red and in blue, respectively).

The geographic coverage of the two datasets includes Europe, Asia, Africa and North America (Fig. 1). In the population-scale dataset, the westernmost site is located in Hawaii, the northernmost in the Netherlands, the easternmost in Japan and the southernmost in Kenya. The majority of the study sites are located in Europe (18 out of a total of 39 locations; Table 1). For the individual-scale dataset, the westernmost site is located in Washington State (USA), the northernmost in France, the easternmost in China and the southernmost in Kenya. As for the population-scale dataset, the majority of the study sites are located in Europe (five out of a total of eight locations; Table 1).

### Design description

Each isotopic record included in the datasets is associated with the corresponding geographical and temporal information of the sampling event including country, location, geographical coordinates (latitude and longitude in decimal degrees), type of habitat and sampling date. In the dataset resolved at population scale, δ^13^C and δ^15^N values of putative prey are specified together with other biological features, such as the invasive or native nature of the species for each location. Stable isotope values can be used for downstream analyses as, for example, the calculation of the trophic position (see Di Muri et al. 2022 for details). In the dataset resolved at individual scale, no information on putative prey is included, as they can be obtained from the population-scale dataset. Isotopic values included in the individual-scale dataset can be used, for example, to estimate isotopic spatial niche metrics, including the convex hull area, the nearest-neighbour distance and the distance to centroid (Jackson et al. 2011, Jackson et al. 2012 after Layman et al. 2007).

### Funding

LifeWatch ERIC Internal Joint Initiative

## Sampling methods

### Study extent

The literature search was concluded on 17 January 2022.

### Sampling description

The Google Scholar engine was used to search for relevant bibliographic sources using the keywords "*Procambarusclarkii*" and "stable isotopes" and a total of 651 publications were returned. Peer-reviewed articles and grey literature material were individually inspected in order to identify the bibliographic sources with stable isotope information of the Louisian crayfish and of its potential prey in tabular or graphical format; 41 studies performed in both freshwater and transitional environments and published between 2005 and 2021 were ultimately selected (Table 1; Suppl. material 1). For data extraction, figures were digitised after a five-fold enlargement and converted to numerical form using the WebPlotDigitizer graph capture software. The quality control of stable isotope data extracted from figures was performed by comparing data available in both numerical and graphical form.

### Quality control

Only records with specified locations were included in the datasets. The location accuracy was checked using Google Earth and, when geographical coordinates were not explicit, the maps of the study sites included in the publications were used to retrieve them. Geographical coordinates were converted to decimal degrees when not originally specified as such. A taxonomic check was additionally performed using the World Register of Marine Species, the GBIF Backbone Taxonomy and the Integrated Taxonomic Information System to check the current accepted scientific names of all taxa included in the datasets.

### Step description

*Procambarusclarkii* is an omnivore species feeding on vegetal (e.g. algae and leaf detritus) and animal prey including invertebrates (e.g. insect larval stages, oligochaetes, gastropods) and vertebrates (e.g. amphibians and fish) depending on resources availability (Alcorlo et al. 2004, Loureiro et al. 2019).

In general, primary producers, including living (e.g. macrophytes, periphyton etc.) and non-living (i.e. detritus) organisms were preferentially used as reference for the selection of the baseline species included in the population-scale dataset. In few instances, herbivorous gastropods, aquatic larval stages of insects and other invertebrates occurring at the study sites and characterised by a trophic position = 2 were chosen (i.e. Reference ID 12 in Suppl. material 1: *Stenopsychemarmorata* [*Shin et al. 2012*]; Reference ID 33 in Suppl. material 1: *Macrobrachiumnipponense* [Zhang et al. 2020]; Reference ID 35 in Suppl. material 1: maggots of *Ephydra* sp. [Bradley and Herbst 1994]; Reference ID 3 in Suppl. material 1: larvae of *Cheumatopsycheanalis* [Zuellig et al. 2004]). Only in two cases, two species of vertebrate predators with a trophic position = 3 were included (i.e. Reference ID 34 in Suppl. material 1: larval stage of the odonate *Epophthalmiaelegans* [Yamada and Urabe 2021]; Reference ID 39 in Suppl. material 1: larval stage of the hydrophilid beetle *Hydrocharaaffinis* [Baek et al. 2014]).

The population-scale dataset additionally includes the sample size of each isotopic record as well as the standard deviations of mean δ^13^C and δ^15^N values. When the standard deviations were not available (i.e. Reference ID 10 in Suppl. material 1), we have used the modified Range Rule to estimate them, as described in Ramirez and Cox (2012) and as reported below:

\begin{varwidth}{50in}
        \begin{equation*}
            \sigma = \frac{\text{range}}{3\sqrt{ln(n)} - 1.5}  
        \end{equation*}
    \end{varwidth}

## Geographic coverage

### Description

The datasets gather isotopic values of *P.clarkii* and its potential prey in invaded lotic and lentic habitats across 10 countries (Table 1). Overall, the study sites were distributed across 27 locations in France mostly located in the Garonne floodplain, nine locations in the USA, seven in Japan, four in China and in Italy, two in Spain and one in Hungary, Kenya, Portugal and the Netherlands.

## Taxonomic coverage

### Description

The dataset resolved at individual scale includes only carbon and nitrogen isotopic values of *P.clarkii*, whereas, the population-scale dataset is a collection of mean isotopic values of *P.clarkii* and its potential prey, including vegetal and animal prey.

### Taxa included

**Table:** 

Rank	Scientific Name	
class	Gastropoda (Cuvier, 1797)	
order	Amphipoda	
order	Ephemeroptera	
family	Asellidae	
family	Chironomidae	
family	Cyrenidae (Gray, 1840) - Corbiculidae in the original publication	
family	Culicidae (Meigen, 1818)	
family	Lymnaeidae (Rafinesque, 1815)	
family	Sialidae	
genus	*Corbicula* (Megerle von Mühlfeld, 1811)	
genus	*Dreissena* (Beneden, 1835)	
genus	*Echinogammarus* (Stebbing, 1899)	
genus	*Ephydra*	
genus	*Gammarus* (J. C. Fabricius, 1775)	
genus	*Myriophyllum* L.	
genus	*Poa* L.	
genus	*Potamogeton* L.	
genus	*Spirogyra* (Link, 1820)	
species	*Alismaplantago-aquatica* L.	
species	*Gabbialongicornis* (Benson, 1842) - *Alocinmalongicornis* in the original publication	
species	*Anodontaanatina* (Linnaeus, 1758)	
species	*Asellusaquaticus* (Linnaeus, 1758)	
species	*Sinotaiaquadrata* (Benson, 1842) - *Bellamyaaeruginosa* in the original publication	
species	*Cipangopaludinachinensis* (Gray, 1833) - *Bellamyachinensis* in the original publication	
species	*Cheumatopsycheanalis* (Banks, 1903)	
species	*Corbiculafluminea* (O. F. Müller, 1774)	
species	*Dreissenapolymorpha* (Pallas, 1771)	
species	*Epophthalmiaelegans* (Brauer, 1865)	
species	*Hydrocharaaffinis* (Sharp, 1873)	
species	*Macrobrachiumnipponense* (De Haan, 1849)	
species	*Menthaaquatica* L.	
species	*Phragmitesaustralis* (Cav.) Trin. ex Steud.	
species	*Pomaceamaculata* (Perry, 1810)	
species	*Potentillaanserina* L.	
species	*Ricciafluitans* L.	
species	*Semisulcospirareiniana* (Brot, 1876)	
species	*Sparganiumerectum* L.	
species	*Stenopsychemarmorata* (Navas, 1920)	
species	*Radixauricularia* (Linnaeus, 1758) - *Radixauriculariajaponica* in the original publication	

## Usage licence

### Usage licence

Creative Commons Public Domain Waiver (CC-Zero)

### IP rights notes

This work is licensed under a Creative Commons Attribution (CC-BY) 4.0 Licence.

## Data resources

### Data package title

Individual and population-scale carbon and nitrogen isotopic signatures of *Procambarusclarkii* in invaded freshwater ecosystems.

### Resource link

https://doi.org/10.48372/d25219d3-fe11-4052-879a-eb2e15ca295c

### Number of data sets

2

### Data set 1.

#### Data set name

Population-scale carbon and nitrogen isotopic signatures of *Procambarusclarkii* in invaded freshwater ecosystems.

#### Data format

csv

#### Download URL

https://dataportal.lifewatchitaly.eu/view/urn%3Auuid%3A18a8256c-0e31-4800-af5b-958ea88faf34

#### Description

A description of the dataset is provided below. Wherever possible, the dataset attributes were labelled using standard vocabularies and terms harvested from Darwin Core, LifeWatch ERIC Ecoportal and NERC Vocabulary Server.

**Data set 1.** 

Column label	Column description
catalogNumber	An identifier (preferably unique) for the record within the dataset or collection.
associatedReferences	A list (concatenated and separated) of identifiers (publication, bibliographic reference, global unique identifier, URI) of literature associated with the Occurrence.
country	The name of the country or major administrative unit in which the Location occurs.
locality	The specific description of the place.
habitat	A category or description of the habitat in which the Event occurred.
eventDate	The date-time or interval during which an Event occurred.
decimalLatitude	The geographic latitude (in decimal degrees, using the spatial reference system given in geodeticDatum) of the geographic centre of a Location. Positive values are north of the Equator, negative values are south of it.
decimalLongitude	The geographic longitude (in decimal degrees, using the spatial reference system given in geodeticDatum) of the geographic centre of a Location. Positive values are east of the Greenwich Meridian, negative values are west of it.
taxonName	Name of the biological entity, taxonomic group or lowest level of taxonomic rank that could be determined.
establishmentMeans	Statement about whether an organism or organisms have been introduced to a given place and time through the direct or indirect activity of modern humans (https://dwc.tdwg.org/em/#dwcem_e).
trophicRole	Statement specifying whether the species is a predator or a prey.
d13C_VPDB_biota	The ratio of carbon 13 relative to carbon 12 in a biological organism identified elsewhere in the metadata, expressed in per mille and relative to the international reference Vienna Pee Dee Belemnite standard.
SD_d13C_VPDB_biota	The square root of the average of the squares of deviations about the mean of a set of values of the specified measurement.
d15N_biota	The ratio of nitrogen 15 relative to nitrogen 14 in a biological organism identified elsewhere in the metadata, expressed in per mille and relative to atmospheric air.
SD_d15N_biota	The square root of the average of the squares of deviations about the mean of a set of values of the specified measurement.
sampleSizeValue_d13C	A numeric value for the measurement of the size (number of samples) in a sampling event for the isotope of the chemical element carbon.
sampleSizeValue_d15N	A numeric value for the measurement of the size (number of samples) in a sampling event for the isotope of the chemical element nitrogen.
trophicLevel	Any of the feeding levels through which the passage of energy through an ecosystem proceeds; examples are photosynthetic plants, herbivorous animals and microorganisms of decay.

### Data set 2.

#### Data set name

Individual-scale carbon and nitrogen isotopic signatures of *Procambarusclarkii* in invaded freshwater ecosystems.

#### Data format

csv

#### Download URL

https://dataportal.lifewatchitaly.eu/view/urn%3Auuid%3A18a8256c-0e31-4800-af5b-958ea88faf34

#### Description

A description of the dataset is provided below. The dataset attributes were labelled using standard vocabularies and terms harvested from Darwin Core, LifeWatch ERIC Ecoportal and NERC Vocabulary Server.

**Data set 2.** 

Column label	Column description
catalogNumber	An identifier (preferably unique) for the record within the dataset or collection.
associatedReferences	A list (concatenated and separated) of identifiers (publication, bibliographic reference, global unique identifier, URI) of literature associated with the Occurrence.
country	The name of the country or major administrative unit in which the Location occurs.
locality	The specific description of the place.
habitat	A category or description of the habitat in which the Event occurred.
eventDate	The date-time or interval during which an Event occurred.
scientificName	The full scientific name, with authorship and date information, if known. When forming part of an Identification, this should be the name in lowest level taxonomic rank that can be determined. This term should not contain identification qualifications, which should instead be supplied in the IdentificationQualifier term.
decimalLatitude	The geographic latitude (in decimal degrees, using the spatial reference system given in geodeticDatum) of the geographic centre of a Location. Positive values are north of the Equator, negative values are south of it.
decimalLongitude	The geographic longitude (in decimal degrees, using the spatial reference system given in geodeticDatum) of the geographic centre of a Location. Positive values are east of the Greenwich Meridian, negative values are west of it.
d13C_VPDB_biota	The ratio of carbon 13 relative to carbon 12 in a biological organism identified elsewhere in the metadata, expressed in per mille and relative to the international reference Vienna Pee Dee Belemnite standard.
d15N_biota	The ratio of nitrogen 15 relative to nitrogen 14 in a biological organism identified elsewhere in the metadata, expressed in per mille and relative to atmospheric air.

## Supplementary Material

87C99A13-5859-55F2-8B31-D2BA766CA9C8 10.3897/BDJ.10.e94411.suppl1 Supplementary material 1 Table S1 Data type Reference list Brief description List of bibliographic references used for isotopic data collection of *Procambarusclarkii* and potential prey. File: oo_670329.pdf https://binary.pensoft.net/file/670329 Di Muri C, Mancinelli G

## Figures and Tables

**Table 1.** List of study sites included in the datasets where isotopic data of *Procambarusclarkii* and its prey were collected. For each study site, information on country, location, habitat, sampling year and associated reference IDs are reported (full reference list in Suppl. material 1). The column "Resolution" indicates whether isotopic data of *Procambarusclarkii* are available at population- or individual-scale.

Country	Locality (no. of sites)	Habitat	Year sampling	Resolution	Reference ID
USA	Nevada, Ash Meadows National Wildlife Refuge (1)	Spring	1999-2000	Population	1
Japan	Lake Biwa Basin (1)	River	2003	Population	2
USA	Hawaii, Hainako stream (2)	Stream	2006	Population	3
France	Garonne River Basin (1)	River	2007-2008	Population	4
Japan	Namegawa (1)	Pond	1999-2000	Population	5
USA	Hawaii, Opaekaa stream (1)	Stream	2008-2009	Population	6
France	Rhone River Basin (1)	Stream	2009	Population	7
Japan	Shizuoka Prefecture (1)	Pond	2009	Population	8
The Netherlands	Lake Terra Nova (1)	Lake	2011	Population	10
Kenya	Lake Navaisha (1)	Lake	2001-2008	Individual	11
Japan	Lake Biwa (2)	Lake	2007	Population	12
China	Lake Chaohu (1)	Lake	2003	Population	13
Spain	Guadalquivir River Basin (3)	River	2000-2001	Individual	14
France	Aquitaine (3)	Lake/River	2009-2010	Population	15
Japan	Asahi River (1)	River	2009	Population	16
USA	Nevada, Ash Meadows National Wildlife Refuge (1)	Spring	2011-2012	Population	17
Japan	Lake Teganuma (1)	Lake	2009	Population	18
China	Lake Gucheng (1)	Pond	2013	Population	19-20
Spain	Ebro River Basin (1)	Reservoir	2006	Population	9-21
Japan	Lake Izunuma (1)	Lake	2006	Population	22
France	Garonne River Basin (15)	Lake	2012	Individual	23
USA	Washington State (5)	Lake	2009	Population	24
Spain	Ebro River Basin (1)	Lagoon	2015-2016	Population	25
Italy	Lake Trasimeno and Lake Bolsena (2)	Lake	2014	Population	26
France	Garonne River Basin (1)	Lake	2014	Individual	27
USA	Washington State (5)	Lake	2014	Individual	27
Italy	Arno River (1)	River	2018	Population	28
Spain	Lake Arreo (1)	Lake	2017	Individual	29-30
France	Garonne River Basin (7)	Lake	2014	Individual	31
Portugal	Quarteira River Basin (2)	Stream	2015	Population	32
China	Huangshui River Basin (1)	Reservoir	2015-2016	Population	33
Japan	Lake Izunuma (1)	Pond	2008	Population	34
Spain	Albufera de València (1)	Marsh	2018	Population	35
Italy	Monterotondo (1)	Pond	2016	Population	36
France	Garonne River Basin (3)	Stream	2019	Population	37
France	Aquitaine (3)	Lake	2015	Population	38
Japan	Tojooka Basin (1)	Pond	2014-2016	Population	39
Hungary	Danube River Basin (1)	Stream	2018	Population	40
China	Lake Dongting (28)	Lake	2017	Individual	41
