# Supplementary material for: Individual and population-scale carbon and nitrogen isotopic values of Procambarusclarkii in invaded freshwater ecosystems
Source: Biodivers Data J. 2022 Oct 20;10:e94411. doi: 10.3897/BDJ.10.e94411 (PMC9836639; doi:10.3897/BDJ.10.e94411)
Supplement: Supplementary material 1 — Table S1 [file bdj-10-e94411-s001.pdf]

Table S1. List of references used for isotopic data collection of *Procambarus clarkii* and potential prey.

ID	References
1	Kennedy TA, Finlay JC, Hobbie SE (2005) Eradication of invasive *Tamarix ramosissima* along a desert stream increases native fish density. Ecological Applications 15 (6): 2072-2083. https://doi.org/10.1890/04-1533
2	Kohzu A, Tayasu I, Yoshimizu C, Maruyama A, Kohmatsu Y, Hyodo F, Onoda Y, Igeta A, Matsui K, Nakano T, Wada E, Nagata T, Takemon Y (2009) Nitrogen-stable isotopic signatures of basal food items, primary consumers and omnivores in rivers with different levels of human impact. Ecological Research 24 (1): 127-136. https://doi.org/10.1007/s11284-008-0489-x
3	Atwood TB, Wiegner TN, Turner P, MacKenzie RA (2010) Potential effects of an invasive nitrogen-fixing tree on a Hawaiian stream food web. Pacific Science 64 (3): 367-379. https://doi.org/10.2984/64.3.367
4	Syväranta J, Cucherousset J, Kopp D, Crivelli A, Céréghino R, Santoul F (2010) Dietary breadth and trophic position of introduced European catfish *Silurus glanis* in the River Tarn (Garonne River basin), southwest France. Aquatic Biology 8 (2): 137-144. https://doi.org/10.3354/ab00220
5	Kobayashi R, Maezono Y, Miyashita T (2011) The importance of allochthonous litter input on the biomass of an alien crayfish in farm ponds. Population Ecology 53 (4): 525-534. https://doi.org/10.1007/s10144-011-0268-3
6	Layhee M (2011) Employing stable isotopes to investigate the impacts of invasive species on Hawaiian stream food webs. California State University URL: https://dspace.calstate.edu/bitstream/handle/10211.3/10211.4_318/Final-Megan%20Layhee.pdf?sequence=1
7	Martino A, Syväranta J, Crivelli AJ, Cereghino R, Santoul F (2011) Is European catfish a threat to eels in southern France?. Aquatic Conservation: Marine and Freshwater Ecosystems 21 (3): 276-281. https://doi.org/10.1002/aqc.1177
8	Miyake, M., & Miyashita, T. (2011). Identification of alien predators that should not be removed for controlling invasive crayfish threatening endangered odonates. Aquatic Conservation: Marine and Freshwater Ecosystems 21 (3): 292-298. https://doi.org/10.1002/aqc.1178
9	Soto DX, Roig R, Gacia E, Catalan J (2011) Differential accumulation of mercury and other trace metals in the food web components of a reservoir impacted by a chlor-alkali plant (Flix, Ebro River, Spain): implications for biomonitoring. Environmental Pollution 159 (6): 1481-1489. https://doi.org/10.1016/j.envpol.2011.03.017
10	van der Wal JEM (2011) Effects of crayfish on the establishment of macrophytes in a shallow peat lake. Netherlands Institute of Ecology (NIOO-KNAW) URL: https://www.stowa.nl/sites/default/files/assets/PUBLICATIES/Publicaties%202012/STOWA%202012-43/NL129%20thesis%20Jessica%20van%20der%20Wal%20crayfish%202011.pdf
11	Jackson MC, Donohue I, Jackson AL, Britton JR, Harper DM, Grey J (2012) Population-level metrics of trophic structure based on stable isotopes and their application to invasion ecology. PloS one 7 (2). https://doi.org/10.1371/journal.pone.0031757
12	Karube ZI, Okada N, Tayasu I (2012) Sulfur stable isotope signature identifies the source of reduced sulfur in benthic communities in macrophyte zones of Lake Biwa, Japan. Limnology 13 (3): 269-280.

https://doi.org/10.1007/s10201-012-0375-7

13 Zhang M, Xie C, Hansson LA, Hu W, Che J (2012) Trophic level changes of fishery catches in Lake Chaohu, Anhui Province, China: trends and causes. Fisheries research 131: 15-20. https://doi.org/10.1016/j.fishres.2012.06.015

14 Alcorlo P, Baltanás A (2013) The trophic ecology of the red swamp crayfish (*Procambarus clarkii*) in Mediterranean aquatic ecosystems: a stable isotope study. Limnetica 32: 121-128. https://doi.org/10.23818/limn.32.12

15 Gentès S, Maury-Brachet R, Guyoneaud R, Monperrus M, Andre JM, Davail S, Legeay A (2013) Mercury bioaccumulation along food webs in temperate aquatic ecosystems colonized by aquatic macrophytes in south western France. Ecotoxicology and Environmental Safety 9: 180-187. https://doi.org/10.1016/j.ecoenv.2013.02.001

16 Kaifu K, Miyazaki S, Aoyama J, Kimura S, Tsukamoto K (2013) Diet of Japanese eels *Anguilla japonica* in the Kojima Bay-Asahi river system, Japan. Environmental Biology of Fishes 96 (4): 439-446. https://doi.org/10.1007/s10641-012-0027-0

17 Kilburn S (2013) Impacts of introduced crayfish on Ash Meadows aquatic communities: Ash Meadows National Wildlife Refuge, Nevada. University of Illinois at Urbana-Champaign URL: http://hdl.handle.net/2142/42342

18 Yamaki A, Yamamuro M (2013) Floating-leaved and emergent vegetation as habitat for fishes in a eutrophic temperate lake without submerged vegetation. Limnology 14 (3): 257-268. https://doi.org/10.1007/s10201-013-0403-2

19 Mao Z, Gu X, Zeng Q (2015) Food sources and trophic relationships of three decapod crustaceans: insights from gut contents and stable isotope analyses. Aquaculture Research 47 (9): 2888-2898. https://doi.org/10.1111/are.12739

20 Mao ZG, Gu XH, Zeng QF, Chen HH (2016) Carbon sources and trophic structure in a macrophyte-dominated polyculture pond assessed by stable-isotope analysis. Freshwater Biology 61 (11): 1862-1873. https://doi.org/10.1111/fwb.12821

21 Soto DX, Benito J, Gacia E, García-Berthou E, Catalan J (2016) Trace metal accumulation as complementary dietary information for the isotopic analysis of complex food webs. Methods in Ecology and Evolution 7 (8): 910-918. https://doi.org/10.1111/2041-210x.12546

22 Yasuno N, Fujimoto Y, Shimada T, Shikano S, Kikuchi E (2016) Ontogenetic dietary shifts of largemouth bass do not increase trophic position in a shallow eutrophic lake in Japan. In Annales de Limnologie-International Journal of Limnology 52: 355-364. EDP Sciences. https://doi.org/10.1051/limn/2016025

23 Jackson MC, Evangelista C, Zhao T, Lecerf A, Britton JR, Cucherousset J (2017) Between-lake variation in the trophic ecology of an invasive crayfish. Freshwater Biology 62 (9): 1501-1510. https://doi.org/10.1111/fwb.12957

24 Larson ER, Twardochleb LA, Olden JD (2017) Comparison of trophic function between the globally invasive crayfishes *Pacifastacus leniusculus* and *Procambarus clarkii*. Limnology 18 (3): 275-286. https://doi.org/10.1007/s10201-016-0505-8

25 Bertolero A, Navarro J (2018) A native bird as a predator for the invasive apple snail, a novel rice field invader in Europe. Aquatic Conservation: Marine and Freshwater Ecosystems 28 (5): 1099-1104.

https://doi.org/10.1002/aqc.2917

26 Mancinelli G, Papadia P, Ludovisi A, Migoni D, Bardelli R, Fanizzi FP, Vizzini S (2018) Beyond the mean: a comparison of trace-and macroelement correlation profiles of two lacustrine populations of the crayfish *Procambarus clarkii*. Science of The Total Environment 624: 1455-1466. https://doi.org/10.1016/j.scitotenv.2017.12.106

27 Evangelista C, Olden JD, Lecerf A, Cucherousset J (2019) Scale-dependent patterns of intraspecific trait variations in two globally invasive species. Oecologia 189 (4): 1083-1094. https://doi.org/10.1007/s00442-019-04374-4

28 Haubrock PJ, Balzani P, Azzini M, Inghilesi AF, Veselý L, Guo W, Tricarico E (2019) Shared histories of co-evolution may affect trophic interactions in a freshwater community dominated by alien species. Frontiers in Ecology and Evolution 355. https://doi.org/10.3389/fevo.2019.00355

29 Haubrock PJ, Balzani P, Criado A, Inghilesi AF, Tricarico E, Monteoliva AP (2019) Predicting the effects of reintroducing a native predator (European eel, *Anguilla anguilla*) into a freshwater community dominated by alien species using a multidisciplinary approach. Management of Biological Invasions 10 (1): 171. https://doi.org/10.3391/mbi.2019.10.1.11

30 Haubrock PJ, Balzani P, Britton JR, Haase P (2020) Using stable isotopes to analyse extinction risks and reintroduction opportunities of native species in invaded ecosystems. Scientific Reports 10 (1): 1-11. https://doi.org/10.1038/s41598-020-78328-9

31 Lang I, Evangelista C, Everts RM, Loot G, Cucherousset J (2020) Stable resource polymorphism along the benthic littoral–pelagic axis in an invasive crayfish. Ecology and Evolution 10 (5): 2650-2660. https://doi.org/10.1002/ece3.6095

32 Sroczyńska K, Williamson TJ, Claro M, González-Pérez JA, Range P, Boski T, Chícharo L (2020) Food web structure of three Mediterranean stream reaches along a gradient of anthropogenic impact. Hydrobiologia 847 (10): 2357-2375. https://doi.org/10.1007/s10750-020-04263-5

33 Yang F, Yu Z, Xie S, Feng H, Wei C, Zhang H, Zhang J (2020) Application of stable isotopes to the bioaccumulation and trophic transfer of arsenic in aquatic organisms around a closed realgar mine. Science of The Total Environment 726. https://doi.org/10.1016/j.scitotenv.2020.138550

34 Yasuno N, Fujimoto Y, Shimada T, Shikano S, Kikuchi E (2020) Unbalanced population structure and reliance on intraspecific predation by largemouth bass in an agricultural pond with no available prey fish. Journal of Freshwater Ecology 35 (1): 523-534. https://doi.org/10.1080/02705060.2020.1853621

35 Antón-Tello M, Britto VO, Gil-Delgado JA, Rico E, Dies JI, Monrós JS, Vera P (2021) Unravelling diet composition and niche segregation of colonial waterbirds in a Mediterranean wetland using stable isotopes. Ibis, 163(3), 913-927. https://doi.org/10.1111/ibi.12928

36 Bissattini AM, Haubrock PJ, Buono V, Balzani P, Borgianni N, Stellati L, Inghilesi AF, Tancioni L, Martinoli M, Tricarico E, Vignoli L (2021) Trophic structure of a pond community dominated by an invasive alien species: insights from stomach content and stable isotope analyses. Aquatic Conservation: Marine and Freshwater Ecosystems 31 (4): 948-963. https://doi.org/10.1002/aqc.3530

37 Garcia F, de Carvalho AR, Riem-Galliano L, Tudesque L, Albignac M, Ter Halle A, Cucherousset J (2021) Stable isotope insights into microplastic contamination within freshwater food webs. Environmental science & technology 55 (2): 1024-1035. https://doi.org/10.1021/acs.est.0c06221

38	Gentès S, Löhrer B, Legeay A, Mazel AF, Anschutz P, Charbonnier C, Tessier E, Maury-Brachet R (2021) Drivers of variability in mercury and methylmercury bioaccumulation and biomagnification in temperate freshwater lakes. Chemosphere 267 https://doi.org/10.1016/j.chemosphere.2020.128890
39	Tawa K, Sagawa S (2021) Stable isotopic analysis of stuffed specimens revealed the feeding habits of oriental storks *Ciconia boyciana* in Japan before their extinction in the wild. Journal of Ornithology 162 (1): 193-206. https://doi.org/10.1007/s10336-020-01806-4
40	Veselý L, Ruokonen TJ, Weiperth A, Kubec J, Szajbert B, Guo W, Ercoli F, Bláha L, Buřič M, Hämäläinen H, Kouba A (2021) Trophic niches of three sympatric invasive crayfish of EU concern. Hydrobiologia 848 (3): 727-737. https://doi.org/10.1007/s10750-020-04479-5
41	Wang Y, Tan W, Li B, Wen L, Lei G (2021) Habitat alteration facilitates the dominance of invasive species through disrupting niche partitioning in floodplain wetlands. Diversity and Distributions 27 (9): 1861-1871. https://doi.org/10.1111/ddi.13376
